# Critique and discussion of “Multicenter evaluation of frequency and impact of activity infiltration in PET imaging, including microscale modeling of skin-absorbed dose”

**DOI:** 10.3389/fnume.2023.1240162

**Published:** 2023-07-31

**Authors:** Josh Knowland

**Affiliations:** Lucerno Dynamics, Cary, NC, United States

**Keywords:** infiltration, extravasation, dosimetry, monte carlo, NRC, nuclear medicine

## 1. Introduction

On May 25, 2023, the Journal of Nuclear Medicine (JNM) published an article ahead-of-print entitled “Multicenter Evaluation of Frequency and Impact of Activity Infiltration in PET Imaging, Including Microscale Modeling of Skin-Absorbed Dose” (1). The paper reports a retrospective study of 1,000 oncology patients from nine positron emission tomography (PET) imaging centers and several smaller clinics. The paper also describes a skin dose calculation using Monte Carlo simulations based on one patient image. For this case, the paper reports that absorbed dose to the skin was approximately 12 mGy.

The paper concludes that the “risk of significant tissue injury from diagnostic PET agents appears negligible, as is consistent with both clinical experience and the literature.” It also asserts that a high level of quality exists in the administration of radiopharmaceuticals in PET practice.

There are, however, several problems with the work that raise questions about its conclusions. The methods are not well described, the results contain errors, and the peer-review process for this manuscript appears to have lacked rigor.

## 2. Methodology problems

For its Monte Carlo simulations, the paper describes an assumed distribution of radioactivity within tissue and skin. However, the distribution is not representative of paravenous extravasation and minimizes the biological effects to muscle.

Extravasated radioactivity is modeled as being contained wholly within the dermis and subcutaneous fat. This assumption is based on previous work involving an injection of dyed saline into porcine subcutaneous fat—an experiment which explicitly excluded muscle. The paper also references “…tumescent fluid injections into the subcutaneous tissue for purposes of local anesthesia are common for several dermatologic procedures, including liposuction, cutaneous surgery, and drug administration.” Subcutaneous administrations are very different than intravenous and are not an appropriate basis for model definition. Very low radiation doses to muscle are reported in Figures 3–5. These results are not explained, but because the underlying model for source activity distribution excludes activity within the muscle itself, absorbed dose may be grossly underestimated.

It is also unclear if the method of estimating initially extravasated radioactivity is appropriate. The paper describes “A measured activity of more than 370 kBq at the injection site, decay-corrected back to time of injection.” This calculation seems to neglect biological clearance and instead corrects for physical decay only. An effective half-life of 30 min is mentioned in later discussions of dosimetry methods, however when estimating the initial activity, the paper makes no mention of biological clearance. Correction of physical decay alone would understate initial activity by nearly 800% for a typical pre-image uptake time of 65 min. Underestimation of initial activity would equally understate reported values for absorbed dose.

The results of the dosimetry method are not compared to existing, widely accepted models, and descriptions of the method lack details needed to replicate the work. Dimensions are provided for volumes of interest, but there is no definition of the material composition or densities used. Furthermore, the text indicates that the cross-sectional dimensions used for Monte Carlo simulation were 36 mm by 21 mm, but Figure 1 states 46 mm by 31 mm. It is not clear which dimensions were actually used for simulation, but absorbed dose for the smaller volume would be nearly double that of the larger.

The paper also states that a total of 1,000 oncology PET studies were analyzed from a variety of imaging centers. These studies were assumed to “…represent the variety of injection skills and injection techniques typically used in the clinical PET environment.” The selection criteria for the imaging centers are not well described. Also, the paper does not provide a statistical justification for the sample size from each institution other than to say that data were from “consecutive patients who had the injection site in the field of view.” The paper does not discuss details such as the number of technologists or their levels of experience.

Figures 2 and 3 also contain discrepancies or values that conflict with the text. In Figure 2, the *x*-axis of subpart A is identified as net activity in kBq. However, the figure's caption states units of MBq. In Figure 3, the *y*-axis is labeled as representing absorbed dose for an example extravasation of 0.83 MBq. The caption, however, references a 0.41 MBq case. Extravasated activities of 0.41 MBq and 0.83 MBq correspond to two different patients presented, but it is unclear which patient is described by Figure 3.

## 3. Discussion

The paper's conclusions seem to start with an assumption that diagnostic radiopharmaceutical extravasations are not a concern, and they should be challenged given the methodology problems identified. The paper states that, “Using the data and assumptions from this work, the risk of significant tissue injury from diagnostic PET agents appears negligible, as is consistent with both clinical experience and the literature.”

The very low tissue doses reported reinforce the belief that injury is unlikely. However, these doses are understated because the model neglects self-dose to muscle and underestimates initially extravasated radioactivity. When an intravenous radiopharmaceutical is extravasated, it will not be confined within subdermal fat. The muscle tissue adjacent to the injection site is valid as both a source and target volume, but it is inappropriately ignored in the paper's dosimetry model. The paper did not provide evidence of validating its dosimetry model against existing published models.

Because the patient's skin is unlikely to be affected by activity near the extravasated vein, any effects to muscle tissue will likely be unnoticed and underdiagnosed by clinicians. Therefore, clinical experience and literature that has not appropriately evaluated underlying tissue injury should not be used to assert that injury from extravasation of diagnostic PET agents is negligible.

The paper also concludes that the rate of clinically meaningful extravasation (1% of injected activity) was between 0% and 0.37%. Inaccuracies in estimation of initially extravasated activity likely caused this rate to be understated. Considering the four cases of extravasation in Figure 2, proper application of biological clearance would cause all four to surpass the defined threshold. Four cases of meaningful extravasation (as opposed to the reported zero) would result in a rate of 0.4% with a 95% confidence interval of 0.11%–1.02%. Even if the extravasation rate reported in this paper were accepted and applied to the 30 million radiopharmaceutical administrations every year in the US (from approximately 20 million nuclear medicine procedures), then up to 111,000 patients may experience clinically meaningful extravasations. Large extravasations have been shown to cause tissue absorbed doses of several Gray (3–6), diminished diagnostic image quality (7–9), and reduced quantitative accuracy (10–12).

The paper also states that “…our data indicate a high level of quality in the administration of radiopharmaceuticals in PET practice.” It is important to note that the results apply only to those institutions, technologists, and PET procedures that were studied. The results from this paper only reflect what happened in these few centers during undefined observation periods and cannot be applied to the practice of nuclear medicine generally. For example, the paper has no description of the training and experience levels of participating technologists, and an unknown number of images with injection sites outside of the field of view were excluded from the study. Additionally, the paper estimates absorbed dose for a hypothetical “complete” extravasation of 470 MBq. It is unclear how this value was chosen, and it is not representative of the maximum injected activity for many nuclear medicine procedures in the US (2). Furthermore, this paper states that the study included no cases where intravenous access was through direct needle stick or butterfly catheter. However, many nuclear medicine centers do continue to use direct needle sticks and butterfly catheters for venous access.

Regarding large extravasations, the paper remarks that, “Instances have clearly been reported in the PET literature…and the field at large must be vigilant.” This is an important point. Neither providers nor patients should worry about extravasations that involve insignificant radioactivity. However, for large extravasations, appropriate steps should be taken to characterize, minimize, and document the event. This paper used 1% of the injected activity as a threshold for clinical meaningfulness. However, tissue absorbed dose is a more appropriate metric for radiation protection of patients. The US Nuclear Regulatory Commission (NRC) has codified radiation exposure levels of concern in 10 CFR Part 35.3045 for medical patients if radioactive material is administered improperly or differently from that which was intended or prescribed. Those criteria include a radiation dose that exceeds 0.05 Sv (5 rem) effective dose equivalent, 0.5 Sv (50 rem) to an organ or tissue, or 0.5 Sv (50 rem) shallow dose equivalent to the skin. For low LET radiation, 0.5 Sv is approximately the same as 0.5 Gy (500 mGy). NRC acknowledges that a radiation dose at or even above these levels may not necessarily result in physical harm to the patient, but rather instances of unintended dose to an organ or tissue may indicate a problem in the medical facility's practice or procedures. Routinely exceeding the 500 mGy threshold should act as an early indicator of increased risk.

Rigorous peer-review would typically address many of the shortcomings and errors identified in this paper. However, this paper seems to have been afforded an unusually accelerated peer-review process. The manuscript was submitted on April 18, 2023, underwent peer-review, and was accepted without revision 8 days later. Examination of other research articles published by the Journal of Nuclear Medicine (JNM) within the past six months reveals that the average time between manuscript submission and acceptance is 142 days (*N* = 106, 95% CI: 132–153 days). The likelihood that this paper experienced JNM's normal review and revision process is low (*p* = 0.0072). An eight-day review time is even less than the average for invited perspectives (*M* = 60 days, *N* = 19, 95% CI: 33–86 days), which are expected to undergo less critical scrutiny.

Only one other recently published JNM article was found to have had unusually accelerated peer-review (12 days): “Adverse Clinical Events at the Injection Site Are Exceedingly Rare After Reported Radiopharmaceutical Extravasation in Patients Undergoing 99mTc-MDP Whole-Body Bone Scintigraphy: A 12-Year Experience” by Parihar, et al. (13). This study evaluated written radiology reports, rather than injection site images, to calculate and report an exceptionally low extravasation rate. The study also reviewed medical records to assess harm, which are unlikely to accurately reflect incidence or causality. An absence of confirmation does not confirm an absence.

It is noteworthy that the JNM is “self-published by the Society of Nuclear Medicine and Molecular Imaging (SNMMI).” The SNMMI has publicly opposed characterizing and reporting extravasations using arguments that are not supported by science or clinical evidence. The papers by Parihar et al., and Sunderland et al., are the only two papers on the topic of extravasation published by the JNM over the last several years and the only two found to have undergone accelerated review. Additionally, on March 2, 2023, an SNMMI leader and member of numerous committees posted on the “SNMMI Connect” members’ forum that the work by Sunderland et al., “was fostered by the SNMMI”. The combination of undisclosed relationships and selective acceleration of the peer-review process results in a diminished likelihood of objective scientific scrutiny and suggests the SNMMI may be unduly influencing JNM editorial staff to support their public position on rulemaking.
